# A structured, journal-led peer-review mentoring program enhances peer review training

**DOI:** 10.1186/s41073-024-00143-x

**Published:** 2024-03-08

**Authors:** Ariel Maia Lyons-Warren, Whitley W. Aamodt, Kathleen M. Pieper, Roy E. Strowd

**Affiliations:** 1 Department of Pediatrics, Section of Neurology; Clinical Care Center, Baylor College of Medicine, Houston, USA; 2 Department of Neurology, University of Pennsylvania Perelman School of Medicine, Philadelphia, USA; 3 American Academy of Neurology, Minneapolis, USA; 4 Departments of Neurology and Internal Medicine, Wake Forest University School of Medicine, Winston-Salem, USA

**Keywords:** Peer review, Research, Surveys and Questionnaires, Mentorship, Training, Journalology

## Abstract

**Background:**

Peer review is essential to the advancement of knowledge. However, training on how to conduct peer review is limited, unorganized, and not well studied. Thus, we sought to determine if a structured mentored peer-review program improved peer review training as measured by multiple quantitative and qualitative assessments.

**Methods:**

This pre-post intervention study enrolled 55 mentees across 5 cohorts from 2020 to 2023. Each cohort completed pre-program evaluations, participated in 2 mentored reviews, and completed post-program evaluations over 6 months. Mentors and mentees completed pre-program demographic and review experience questionnaires. Outcome measures included (1) total and sub-scores on the modified Review Quality Index (mRQI) applied to the same pre-selected research manuscript reviewed by mentees both pre and post intervention, (2) mentee self-perceived comfort with and understanding of the review process using a custom questionnaire, and (3) mentor satisfaction surveys. Pre- and post-program measures were compared using the Wilcoxon signed-rank test.

**Results:**

Post-program total modified RQI score (median (IQR) = 31 (26.3–35.8)) was higher than pre-program total score (26.6 (19.7–29.7)) for the 42 mentees who completed both pre- and post-program reviews. Mentees reported improved perception of review (median (IQR) pre = 4 (3–4), post = 5 (4–5)) and editorial processes (pre = 3 (2–4), post = 4 (4–5)) as well as self-perceived confidence in completing an independent review of both scientific (median (IQR) pre = 2 (2–3), post = 4 (4–4)) and non-scientific (pre = 3 (2–4), post = 4 (4–5)) manuscripts following program participation. *p* < 0.0001 for all scores noted. Mentors reported high scores for enjoyment (median (range) 5/5 (3–5)) and interest in repeat participation (5/5 (2–5)).

**Conclusions:**

A 6-month structured mentored-review program including 2 mentored reviews improves peer review training as measured by the modified RQI as well as participant self-perceived understanding of publication science with high mentor satisfaction.

**Supplementary Information:**

The online version contains supplementary material available at 10.1186/s41073-024-00143-x.

## Background

Peer review is an essential part of the scientific publishing process to ensure that high quality, methodologically rigorous, peer-vetted work advances a given field. During review, an author’s scientific, research, or scholarly ideas are subjected to the scrutiny of others who are experts in the same field [1]. This process serves as a filter, assessing the quality of scientific literature as well as the integrity and authenticity of the research itself, although it is not a perfect fraud detector [2]. Recent initiatives such as PubPeer, a website that allows users to review published manuscripts, extends the peer-review process into the post-publication realm as well [3]. Despite the existence of scientific peer review since the 18^th^ century, there is no single program, structure, or onboarding process for developing a trained reviewer pool that has been consistently implemented across or within journals. Further, individual programs have not been reliably validated [4]. Now more than ever, integrity and authenticity in scientific publication is critical. Rapid publication models, pre-print servers, and expedited review have been critical for informing the public of major advances and health concerns in crises such as the COVID-19 pandemic [5, 6]. However, rapid dissemination of information and open artificial intelligence platforms have also highlighted problems with misinformation, lack of reproducibility, and duplication of studies [7]. High quality peer review is one way to ensure scientific integrity but requires that reviewers have (1) sufficient content knowledge, (2) the ability to critically appraise scientific study, (3) effective written communication skills, and (4) an understanding of the editorial process and purpose [8–10].

Despite its importance in the field of medicine, the process of learning how to review a manuscript is largely informal and not a required component of medical training [11]. Journals often call upon reviewers based on their content expertise; however, there are limited resources to train reviewers in critical appraisal, effective communication, and journalology. Further, one study suggested 20% of potential reviewers completed up to 94% of reviews, suggesting a shortage of engaged reviewers [12–15]. This limited pool increases the existing burden on reviewers to review more papers, on editors to detect errors or study flaws, on journals to ensure the timeliness of editorial decisions,, and on authors to allow for fairness and transparency. Thus, there is a need for interventions to prepare early career physicians and scientists to participate in peer review and thus increase the pool of available, qualified reviewers without increasing time burdens on current reviewers [11, 16].

A scalable, sustainable, effective program to teach both physicians and scientists intraining to conduct quality peer review would address these important gaps. Prior randomized studies of trainees [17] and new reviewers at a single journal [18] failed to demonstrate improvements in review quality, error identification, or knowledge. In addition, a systematic review and meta-analysis of 7 interventions to improve peer review concluded that training did not improve the overall peer review quality [19]. Three potential explanations included (1) high levels of motivation among control participants that led to self-improvement, (2) high baseline participant reviewer ability in both control and intervention groups based on enrollment criteria, and (3) the inherent difficulty of teaching peer review assessed over a relatively small number of interventions. In a more recent Cochrane review evaluating grant and peer reviewer training interventions, there was also low-certainty evidence that reviewer training 1) slightly improved a peer reviewer’s ability to detect errors and 2) had little or no effect on assessment of peer review quality compared to standard journal practice, warranting further studies that use a broader spectrum of outcome measures [19]. To address these limitations and determine whether a structured, journal-led peer-review mentoring program could improve the quality of peer review training, the Resident and Fellow Section (RFS) of *Neurology* launched a Mentored Peer Review Training Program in 2010. Between 2010–2019, the program structure was piloted, a formal curriculum was developed for mentors, and written materials were generated to guide novice peer reviewers [20]. In 2020, we set out to formally evaluate the effectiveness of this program. The study was not pre-registered. Here, we aim to show that participation in a mentored peer review program improves peer review training as measured by pre and post program scores on (1) a modified version of the Review Quality Index (RQI) to assess review quality [21], (2) a mentee questionnaire to assess self-perceived comfort and understanding of the review process and (3) a mentor satisfaction survey to assess program sustainability.

## Methods

### Study design

**Fig. 1:**
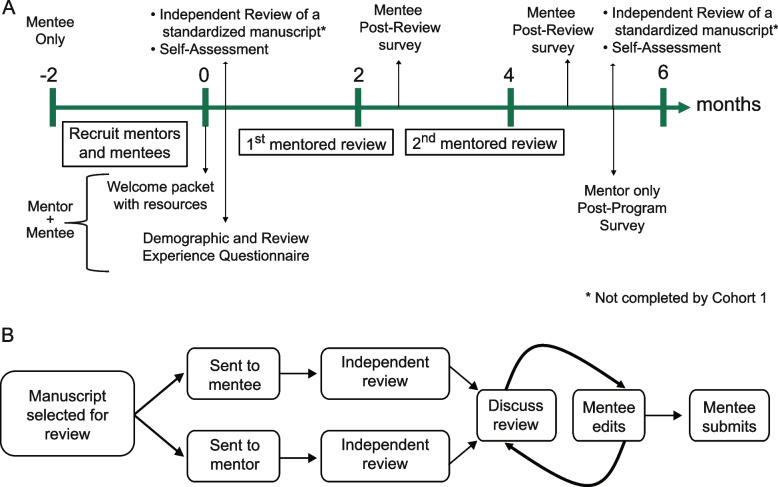
Schematic of Program Structure. **A** Timeline in months for each cohort of participants from recruitment through post-program assessments. **B** Flowchart illustrating mentor–mentee workflow after manuscripts are assigned

Beginning in September 2020, mentees and mentors were enrolled in the Mentored Peer Review Training Program across five cohorts with each cohort taking approximately six months to complete the program (Fig. 1). At the start of the program, each mentor–mentee pair received a welcome packet including a pre-program demographic and review experience questionnaire, resources on how to review a manuscript (supplement) and a description of the program timeline (Fig. 1A). The mentee questionnaire included a self-assessment section. For cohorts two through five, mentees were asked to complete an independent review of a pre-selected manuscript (the same manuscript was reviewed by all participants). They were not told the review would be scored. The pre-selected manuscript, taken from Biorxiv, described a prospective study of stroke incidence before and after the arrival of COVID-19 in a hospital in Bangladesh. This pre-print was subsequently published in *PLoS One* [22].

Mentors and mentees were required to sign up as reviewers for *Neurology*. Case-based manuscripts submitted to the RFS considered appropriate for review by an editor were assigned to the mentor–mentee pairs on a rolling basis. A shared Google spreadsheet was used to track manuscript assignment, review request date, and review receipt date. Assigned manuscripts were sent to the mentor and mentee separately who began with an independent review and later met to discuss their findings. The mentor–mentee meetings were self-coordinated by the pair. Once the mentor felt the review was ready, the mentee submitted the joint review through the reviewer portal. (Fig. 1B) Mentored reviews were not evaluated.

Once all mentor–mentee pairs within each cohort had completed their first mentored review, a Post-Review survey was sent to mentees (supplement) and then the process was repeated for a second mentored review. After all pairs had completed their second review, mentors completed a post-program satisfaction survey and mentees completed self-assessment questions (supplement). For cohorts two through five, mentees also submitted an independent review of the same pre-selected manuscript they had reviewed as part of the pre-assessment.

The a priori hypothesis was that mentored review would improve peer review quality as measured by the mRQI. The study was not preregistered.

### Participants

Mentees were all neurology residents or fellows at the time of acceptance to the program. Three cohorts were comprised of individuals who had applied for a position on the *Neurology* Resident and Fellow Editorial Board. For these cohorts, applicants accepted to the board were enrolled in the program to support them in their new editorial role. Additional applicants considered strong editorial board candidates but not accepted to the board were sent an e-mail invitation to participate in the program. All mentees accepted participation. The other two cohorts were recruited via an open call for applications announced on a *Neurology* blog and publicized over social media [23]. For these cohorts, applicants submitted their name, preferred e-mail address, current institution, year in training, number of manuscripts reviewed (for *Neurology* and for other journals), two to three sentences on why they were interested in this program, and a statement that they had the support of their residency/fellowship director and/or chair to commit ~ 3 h/month to this program. ALW reviewed all applications and selected candidates based on high interest but limited access to mentorship in this area. Selected candidates were offered a spot in the program via e-mail.

Mentors were recruited from known adult and child neurology academicians with a track record of well written reviews for the *Neurology* journal or experience in neurology education. Mentors responded to a blog post [23] via e-mail or were approached directly by e-mail from ALW. Nine mentors participated twice and four mentors participated three times across the five cohorts. Returning mentors did not repeat assessments (Fig. 1). Mentors and mentees were matched based on neurology areas of interest.

Of note, the program requires a program coordinator committing up to two hours per month for 15 participants.

### Measurement tools

Four assessments were used in this study as follows:

#### Demographic and review experience questionnaires

Prior to being matched, mentors and mentees completed a pre-assessment developed by the authors for this study (supplement) which asked them to report their demographic information (name, age, gender, advanced degrees, years since medical school graduation, current level of training), review experience (frequency of reading scientific journals, participation in scientific research, number of published articles, amount of peer review experience, and access to a mentor), and three goals related to participation in the program. The mentee version of this survey also included four questions about comfortability with the review and editorial process (self-assessment). Self-assessment questions were administered both pre and post program participation. Responses were recorded on a 5-point Likert scale in which higher numbers indicated greater understanding.

#### Modified version of the Review Quality Index

The second assessment was a modified version of the Review Quality Index (mRQI) used to evaluate quality of the independent (non-mentored) pre and post program review of a pre-selected manuscript. Only the independent reviews written by mentees were scored. Reviews generated as part of the intervention were not scored. The Review Quality Instrument (RQI) is a validated tool that examines the extent to which a review comments on five aspects of a manuscript (i.e., importance of the research question, originality of the paper, strengths and weaknesses of the method, presentation, interpretation of results) and two aspects of the review (i.e., constructiveness and substantiation of comments) [2]. It has been used previously to study the quality of peer review and impact of training interventions [17]. Importantly, the RQI measures only if a domain is present in the review, not if the reviewer’s assessment of that domain is an accurate reflection of the manuscript. Importantly, researchers have raised concern that the RQI is not an optimal measuring tool [24], as it only reflects *if* reviewers address specific domains, not the accuracy of comments. The mRQI differs from the original review quality index because it also assesses organizational components of a review and measures reviewer appraisal of references. The mRQI has 14 questions. Questions 1–4 (supplement) were scored 0 (absent) or 1 (present), and questions 5–14 were scored on a 5-point Likert scale with higher scores indicating better evaluation of a given review element. The additional questions (Q1-4 and Q12) added to the RQI were developed by the authors. Total mQRI score was calculated by summing the score for each question. Specifically, questions 1–4 were scored 0 (no) or 1 (yes) and questions 5–14 were scored 1–5 on a Likert scale. Thus, the minimum possible score was 10 and the maximum possible score was 54. Eleven participants did not complete the pre-program independent review and two additional participants did not return the post-program independent review. There were no questions about the experience of reviewing the same (pre-selected) manuscript twice.

#### Mentee post-mentored review survey

The third assessment was a brief questionnaire developed by the authors to measure quantitative and qualitative metrics of the mentored review experience (supplement). It was administered to each mentee twice, once after each completed mentored review (Fig. 1a). It included two open-ended questions: “What is one thing you learned from this mentored review” and “How will this experience change your approach to reviewing a manuscript.”

#### Mentor post-program survey

The fourth assessment was a 5 question post-program mentor survey (supplement) in which they were asked to rate their enjoyment of the program and willingness to participate again on a 5-point Likert scale, with 5 indicating strong agreement. They were also asked to provide free text comments on program barriers and benefits. The two most common themes were identified from each open-ended question.

### Statistical methods

Three unblinded independent reviewers (ALW, WWA, RES) scored pre- and post-program independent reviews using the mRQI. Individual and total question scores from each independent reviewer were averaged to obtain final scores. Wilcoxon signed-rank tests were used to compare scores for the 42 participants who completed both pre- and post- program independent reviews. By using pre- and post-program evaluations, each participant served as their own internal control. To assess within-rater reliability, we calculated an R^2^ correlation for total score on mRQI obtained by summing the responses to all questions of the mRQI with the mean score for question 14, “What is the overall quality of the review”. To measure inter-rater reliability, we calculated the intraclass correlation coefficient (ICC) for total mRQI score from pre- and post-program reviews. Wilcoxon signed-rank tests were used to evaluate pre- vs post-program responses to the four questions on assessment of understanding and comfortability with the review process. Open-ended survey responses were coded by author WWA and reported by frequency according to major theme. Statistical tests were performed in Prism (RRID:SCR_002798).

The full dataset including statistical output scripts were shared with the journal during external review. De-identified data is available upon request but was not deposited in a public repository given that mentor names are already publicly available, making it difficult to ensure anonymity amongst the small group.

This study was evaluated by the institutional review board at Wake Forest University and determined to be exempt from requirement for individual authorization as all information was de-identified. Exempt protocol #IRB00097410. Participants were aware that the program was being evaluated but the specific study goals were not shared, and all data were deidentified prior to analysis. Neither patients nor the public participated in method development, data collection nor data analysis.

## Results

### Participants

From September 2020 through March 2023, 55 mentees (31 (56%) female, median age 31 years, eight (14.5%) international) and 38 unique mentors (13 (34%) female, median age 38.5 years) were enrolled in the Mentored Peer Review Training Program across five cohorts with each cohort taking approximately six months to complete the program (Fig. 1, Table 1). 30 mentors returned their pre-program demographic survey.

**Table 1:** Participant demographics

	**Mentees (*n* = 55)**	**Mentors (*n* = 38)**^a^
Female, *n* (%)	31 (56%)	11 (33%)
Age, median (interquartile range) years	31 (29–33)	38.5 (34.25 -42)
Resident & Fellow Section (RFS) board, n (%)	27 (49%)	21 (54%)
Advanced Degrees, n (%)		
Doctor of Medicine (MD) only	35 (64%)	19 (50%)
Doctor of Osteopathic Medicine (DO) only	1 (2%)	0
+ Doctor of Philosophy (PhD)	9 (16%)	13 (34%)
+ Master of Public Health (MPH)	2 (4%)	2 (7%)
+ Master of Sciences (MSc)	5 (9%)	6 (16%)
+ other (Bachelor of Medicine [MBBS], Doctor of Medicine [DM], Master of Arts [MA], Master of Education [MEd])	2 (4%)	1 (3%)
Current Post-Graduate Year, n (%)		
1	3 (5%)	
2	2 (4%)	
3	33 (60%)	
4	9 (16%)	
5	5 (9%)	
6 or greater	3 (5%)	
Years post residency, mean ± std	-	6.03 ± 4.1

^a^Sex and RFS board status were known for all mentees and mentors. *N* = 30 for mentor age

Participants included residents and fellows with a mix of exposure to peer review prior to participation (Table 2) divided into five cohorts ranging from nine to 14 participants (Table 1). While distribution of advanced degrees was similar between groups (Table 1), mentors read journals more frequently and had more experience reviewing manuscripts than mentees (Table 2).

**Table 2:** Mentors read journals and review manuscripts more frequently than mentees

	**Mentees (*n* = 55)**	**Mentors (*n* = 29)**
How often read journals		
Daily, n (%)	9 (16%)	13 (46%)
Weekly, n (%)	28 (51%)	15 (54%)
Monthly, n (%)	15 (27%)	1 (< 1%)
Less than once a month, n (%)	3 (5%)	0
Number of manuscripts reviewed in the last year		
< 2, n (%)	29 (53%)	0
2–5, n (%)	15 (27%)	7 (24%)
6–10, n (%)	2 (4%)	8 (28%)
> 10, n (%)	9 (16%)	14 (48%)

### Validation of the mRQI

**Fig. 2:**
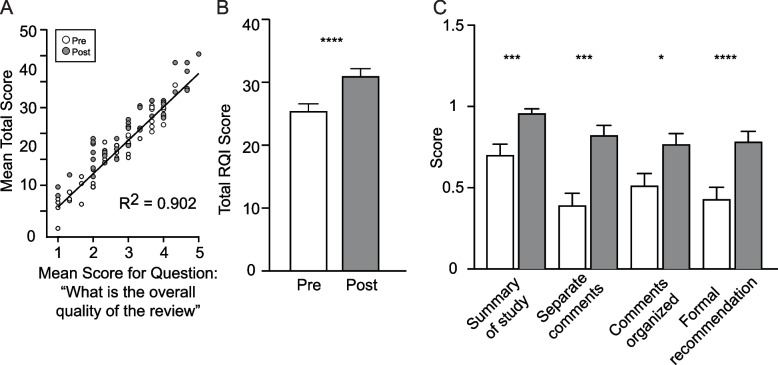
Quantitative assessment of review quality using the modified RQI. Scatter plot (**A**) comparing mean total score and mean score for question 14 (overall quality of the review) for both pre (white dots) and post (gray dots) program reviews demonstrating high correlation. Average total (scores on the modified RQI (**B**) and for questions 1–4 (**C**) on the modified RQI for pre (white bar) and post (gray bar) program reviews. Questions 1–4 were scored Yes = 1 or No = 0 based on presence of absence of each structural component

Total mRQI score was highly correlated (R^2^ = 0.902) with the mean score for question 14, “What is the overall quality of the review” (Fig. 2A), suggesting total mRQI captured the reviewer’s overall assessment of review quality. Inter-rater reliability was also high, and there was strong agreement for total mRQI score from pre-program (ICC 0.786 [CI 0.649–0.880]) and post-program (ICC 0.861 [CI 0.765–0.925]) reviews.

### Improvement in mRQI

42 participants completed both pre- and post- program independent reviews. Total mRQI score pre- and post-program participation (Fig. 2B) showed significant improvement (pre-program median score 26.6 (19.7–29.5), post-program median score 31 (26.3–35.8), *N* = 42, Wilcoxon signed-rank test *p* < 0.0001). Out of the 14 individual items on the mRQI, nine were statistically different (Table 3). Considering the structural elements of the review, we saw a significant increase in the likelihood of including a summary of the study at the beginning of the review, structuring the review with separate comments for authors and editors, using an organizational system such as providing comments by manuscript section, and including a formal recommendation ( Fig. 2C, Table 3).

**Table 3:** Mean Pre and Post Program mRQI Individual Question Scores

Question #	Question Topic	Pre	Post	*P* value
4	Formal recommendation	0.4	0.8	< 0.0001
11	Comment on interpretation of results	2.1	2.8	< 0.0001
2	Separate comments for editor	0.4	0.8	0.0001
1	Summary of the study	0.7	1.0	0.0003
7	Strengths/weaknesses of methods	2.6	3.1	0.0003
9	Comments constructive	2.8	3.4	0.0008
14	Overall quality of review	2.6	3.1	0.0027
6	Originality of paper	1.8	2.2	0.0040
3	Comments divided/organized	0.5	0.8	0.0135
10	Substantiate comments with examples	2.7	3.0	0.1114
5	Importance of research question	1.9	2.3	0.1765
8	Specific, useful comments on writing, organization, figures	2.6	2.8	0.2695
13	Overall tone	3.6	3.6	0.3547
12	Appropriateness of references	1.3	1.4	0.6313

Similarly, we found that comments were viewed as more constructive (Q9), more likely to discuss essential components including strengths and weaknesses of the method (Q7), author interpretation of results (Q11), and originality of the paper (Q6). In contrast, post-program reviews did not improve in their discussion of the research question (Q5), use of specific examples to substantiate comments (Q10), or discussion of appropriateness of references (Q12). Finally, we did not see any improvement in the likelihood of including specific, useful comments on writing, organization, tables, and figures (Q8) or the overall tone of the comments (Q13) likely related to the high pre-program scores in these areas (Table 3).

### Self-perceived improvement in understanding of publication science

**Fig. 3:**
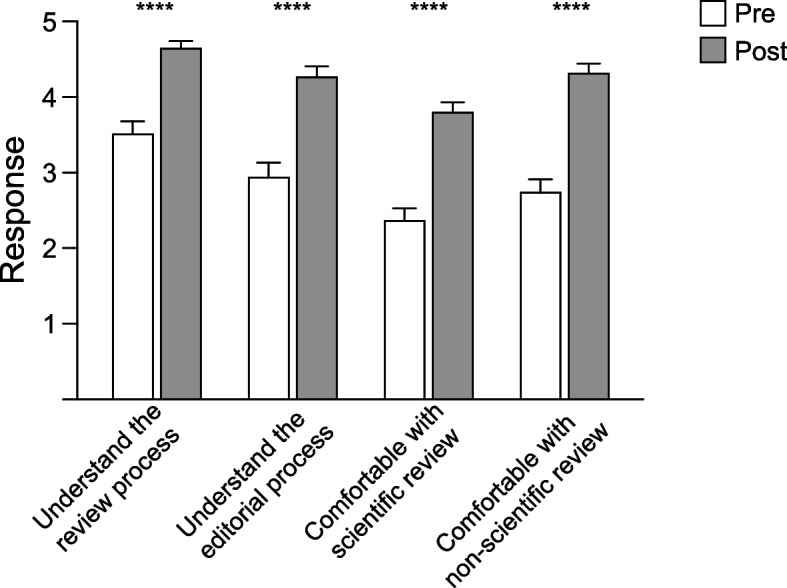
Participant’s reported subjective improvement in reviewing skills. Pre (white bars) and post (gray bars) average response to each of 4 questions posed to each participant before and after program participation. Responses options were 1 (strongly disagree) through 5 (strongly agree)

Next, we assessed impact on participant self-perceived understanding of the review and editorial process. 54 mentees from cohorts one through five completed both pre- and post-program survey questions (supplement). Participant perception of understanding the review (median (IQR) scores pre = 4 (3–4), post = 5 (4–5)) and editorial processes (pre = 3 (2–4), post = 4 (4–5)) significantly increased. In addition, confidence in completing an independent review of both scientific (median (IQR) scores pre = 2 (2–3), post = 4 (4–4)) and non-scientific (pre = 3 (2–4), post = 4 (4–5)) manuscripts significantly increased following program participation (Wilcoxon signed-rank tests, all *p* < 0.0001) (Fig. 3).

### Self-perceived improvement in critical appraisal skills

42/44 mentees from cohorts one through four provided answers to post-review survey questions. We reviewed their qualitative comments and identified the two most common themes for each question. When asked to describe one skill they learned after completing the first mentored review, 19/42 (45.2%) mentees reported overall improvement in their ability to structure and organize comments to editors and authors, and 13/42 (31.0%) mentees learned to provide more constructive feedback. After completing their second review, 16/42 (38.1%) mentees again reported improvement in their ability to provide constructive feedback to authors, especially in a more targeted, concise manner with proper tone, while 8/42 (19.1%) mentees reported improvement in review structure. When asked to discuss how they will change their approach to future manuscripts after the first mentored review, 4/42 (9.5%) mentees planned to be more mindful of a manuscript’s suitability for the target audience, and 4/42 (9.5%) mentees commented on the importance of considering a manuscript’s novelty and contribution to the broader literature. After the second mentored review, 7/42 (16.7%) planned to improve the quality of feedback to authors, while 4/42 (9.5%) emphasized the importance of addressing a manuscript's overall value. One mentee wrote:

*“Look at the big picture: consider the value of the manuscript, what it’s supposed to teach us, and whether it is accomplishing that goal. Is the manuscript even necessary or is it teaching us something we already know? Do not ignore the references.”*

### Program satisfaction

Finally, 23/38 (60.5%) mentors completed a post-program assessment (supplement). Median (range) scores for enjoyment and repeat participation were 5/5 (3–5) and 5/5 (2–5), respectively, indicating high levels of satisfaction. When asked to describe benefits to participation, 13/23 (56.5%) felt the program made them stronger peer reviewers and 5/23 (21.7%) felt their involvement developed their skills as mentors and educators. When asked if they would like to provide feedback on program participation, one mentor wrote:

*“Mostly that I enjoyed it! But I also think it helps me become a better reviewer, too, to review the process and the specifics with a mentee.”*

For 13/23 (56.5%) mentors, the main barrier to participation was time with few other reported barriers. Lastly, when asked to identify an area of program improvement, both mentees (2/43; 4.7%) and mentors (6/23; 26.1%) asked to review scientific manuscripts rather than solely critique case reports, as scientific manuscripts are more complex and ubiquitous in academic medicine.

## Discussion

We have demonstrated that participation in a mentored peer review program improves (1) review quality as measured by a modified version of the Review Quality Index and (2) self-perceived critical appraisal skills and understanding of the review process with high mentor satisfaction. Further, this program was easily integrated into an existing journal infrastructure without adding significant time or workload to journal staff, suggesting a small change in editorial infrastructure could broadly impact how we train reviewers, creating a pipeline to increase the pool of available, qualified peer reviewers.

Our results build on important prior work in the area of peer review. At baseline, peer review is difficult to perform as it is not an innate skill, and approaches to reviewing an article are not systematically taught nor are they included in accreditation standards for biomedical training in the US or internationally [25–27]. What defines a “good” review is also difficult to standardize or even articulate and can vary depending on the type of manuscript, goals of the review, and audience of the journal [28].

Prior approaches to training peer reviewers can be divided into mentored vs. non-mentored training programs, single vs. longitudinal peer review workshops, and self-taught vs. guided instruction [25]. Journal level initiatives to improve the quality of reviews, such as checklists, open peer review, group review, and blinded review have also been tried. Yet very few of these methods have been proven to enhance the value of a review [29, 30]. Indeed, the only randomized controlled trial testing the effectiveness of mentored peer review training in a journal setting failed to show improvement in review quality as measured by the journal’s internal review rating scale [18, 31]. We suggest the lack of improvement in this trial was due to the high baseline ability of participants who were selected based on prior publication and review experience. In contrast, our program targets early career individuals with limited to no experience. To effectively evaluate a manuscript, we propose that a reviewer must have (1) content expertise, (2) critical appraisal skills, (3) effective written communication skills, and (4) understanding of publication science. Topics such as fraud detection, post-publication review, and others are also important skills and may be foundational to obtain prior to such a program, though this was not evaluated in the current study. We found that our mentored review program improved reviews on measures of publication science, critical appraisal, and structure but not areas related to content knowledge. This distinction represents an important nuance of peer review that merits further investigation and may explain the lack of improvement seen in prior studies for which most participants already had expertise in items 1–3. To have content expertise, a reviewer needs to fully understand the science, have clinical or research experience providing appropriate perspective as well as intellectual humility to recognize gaps in their knowledge. Our data suggest that these skills cannot be directly taught but are learned over time. However, this leads to the provocative question: do individuals with training in the general review process develop critical thinking in their content area sooner, or more effectively communicate constructive criticism once they have gained sufficient content knowledge? If so, training in “basic” review structure could be added to all medical school curricula. If not, journals should prioritize developing review resources for content experts.

In contrast to content expertise, our data suggest that skills required for critical appraisal of a manuscript, including critical thinking, curiosity, and experience with evaluation can be readily taught. The themes identified by our participants in their post-review surveys articulate these skills which were developed from conversations around a mentored review rather than specifically articulated through program resources. The greatest areas of improvement in our cohorts were the development of self-perceived scholarly communication skills and self-perceived understanding of publication science. Notably, our participants experienced this increase in skills following only two mentored reviews, suggesting these foundational skills could be easily incorporated into training programs. These improvements were seen across participants with a wide variety of editorial experience and backgrounds ranging from editorial board members to a medical intern.

### Study strengths

The strengths of this study include the cohort size, use of multiple metrics for evaluating improvement, and variable pre-program skill level of mentees which likely reflects the overall reviewer population. Further, a major strength of this program was integration with a journal’s established peer review system demonstrating a process by which peer review training can be implemented without excessive burden on journal staff, editors, or mentors. While we only report two and a half years of pre-post program data here, our program is ongoing and proves that mentored peer review programs built into existing journal infrastructure are sustainable, similar to non-mentored learning repositories.

### Study limitations

This study also has several limitations. Most importantly, the mRQI only assesses the presence or absence of domains within the review, not if the reviewer’s comments are accurate [24]. Thus, we cannot conclude that post-program reviews more accurately evaluated the test article. Future studies could improve upon this analysis by creating a list of expected crucial points an experienced reviewer would identify or comparing participant reviews to real-life peer reviews, both of which would allow more sensitive and specific evaluation of review quality. However, we feel that review quality encompasses many aspects of a review, including structure, awareness of what aspects of the paper to evaluate, and tone. Similarly, we did not evaluate intermediate mentored reviews because the final review received by the journal was the result of multiple rounds of input from the mentor and it was therefore impossible to know which components came directly from the mentee. In the future, we could ask the mentee to submit their first version of the review and the mentor to submit the final version and compare the two. Next, the RFS only publishes case-based articles, yet our pre- and post-program metric was based on a research article. Thus, lack of improvement in some areas might reflect lack of opportunity to discuss research methodology with the mentor. It is possible that a mentored review program incorporating manuscripts of different types might be more impactful than the structure reported here. Along these lines, this was not a randomized controlled trial. Thus, we do not know how trainees at this level, reviewing for the RFS, would have improved with a simpler intervention. Similarly, improvement might be due to participants re-reading the same article a second time. Use of a single manuscript with a finite set of errors also limits the generalizability of our findings. Finally, there is a risk of rating bias as the evaluators were not blinded.

## Conclusions

We show that a two-review, integrated mentorship program improved the structure of submitted reviews which reflects new skills related to writing as a reviewer. Further, this program helps increase confidence in reviewing, likely due to increases in understanding the publication process and purpose of a review. However, this program is not a panacea. We still need reviewers with content knowledge and methods to teach critical appraisal. While there are indications that mentored review can support this process, developing these skills requires additional training and investment in physicians as reviewers and scientists.

### Supplementary Information

**Supplementary file 1. **

## Data Availability

The datasets used and/or analyzed during the current study are available from the corresponding author on reasonable request.

## Abbreviations

RFS: Resident and Fellow Section
RQI: Review Quality Index
mRQI: Modified Review Quality Index
