# Supplementary material for: A structured, journal-led peer-review mentoring program enhances peer review training
Source: Res Integr Peer Rev. 2024 Mar 8;9:3. doi: 10.1186/s41073-024-00143-x (PMC10921741; doi:10.1186/s41073-024-00143-x)
Supplement: Supplementary file 1 [file 41073_2024_143_MOESM1_ESM.docx]

**Online Supplement**

Lyons-Warren AM, Aamodt WW, Pieper KM, Strowd RE. A structured mentored review program improves the quality of peer review: a pre-post intervention study. *Research Integrity and Peer Review* 20xx;x:x. https://doi.org/xxxxxxxxxx.

Table of Contents

[Mentee Survey 2](#_Toc140487720)

[Welcome Packet and Instructions 4](#_Toc140487721)

[Introduction Letter 4](#_Toc140487722)

[Additional Resources for Peer Review 5](#_Toc140487723)

[Structuring a Review 6](#_Toc140487724)

[A note on tone 6](#_Toc140487725)

[Factors to consider when evaluating a manuscript 6](#_Toc140487726)

[Appendix 1: Sample template - Comments for the editors 8](#_Toc140487727)

[Appendix 2: Evaluating your review 9](#_Toc140487728)

[Mentor Evaluation of Reviews 10](#_Toc140487729)

[Mentee First Review Follow-up Survey 11](#_Toc140487730)

[Mentee Second Review Follow-up Survey 12](#_Toc140487731)

[Mentor Survey regarding Mentor/ Mentee Program 14](#_Toc140487732)

[Modified Review Quality Index 14](#_Toc140487733)

# Mentee Survey

**Thank you for agreeing to be a MENTEE in the Resident & Fellow Section of Neurology®. The following questions will help us to develop and optimize this program for our mentors and your fellow mentees. We ask that you please complete the following short questions which we will use as we plan, prepare, and assess the value of this opportunity. Thank you in advance for your participation.**

Name: Date:

1. Age:

2. Sex:  M  F  Nonbinary  Prefer not to say

3. Advanced degrees  MD  DO  PhD  MPH/MHS  MSC  Other:

4. Years since med school graduation:

5. Current level of training: PGY  3  4  5  6  7  Other:______

6. Do you routinely read any scientific journals?

Daily  Weekly  Monthly Less than once a month  Not at all

7. Have you participated in scientific research?

Yes  No

8. Have you published any peer-reviewed manuscripts?

0  1  2-5  6-10  >10

9. Have you participated in peer review?

Yes  No

9b. If you have participated in peer review, please list the journals for which you have reviewed manuscripts:

___________________________________________________________

9c. If you have participated in peer review, how many manuscripts have you reviewed?

0  1  2-5  6-10  >10

9d. If you have participated in peer review, have you had a mentor or have you performed your review(s) in combination with a more senior colleague in the field?

Yes  No

10. Do you currently have a faculty mentor?

Research mentor  Career mentor  Do not have a mentor  Other _____

Please answer the following questions by selecting a rating:

11. I understand the review process involved in peer review of scientific manuscripts:

1  2  3  4  5

Strongly disagree Disagree Neutral Agree Strongly agree

12. I understand the editorial process involved in processing a manuscript for publication:

1  2  3  4  5

Strongly disagree Disagree Neutral Agree Strongly agree

13. I am confident that I can independently review a SCIENTIFIC manuscript at a high level:

1  2  3  4  5

Strongly disagree Disagree Neutral Agree Strongly agree

14. I am confident that I can independently review a NON-SCIENTIFIC (i.e. clinical) manuscript at a high level:

1  2  3  4  5

Strongly disagree Disagree Neutral Agree Strongly agree

As with any new experience, you hope to gain something from this opportunity with the Resident & Fellow Section of Neurology®. Please list the TOP 3 GOALS that you will set for this mentor-mentee experience in peer-review of manuscripts for the Resident & Fellow Section. Be as specific as possible (you may list more than 3 if you would like):

Goal 1:

Goal 2:

Goal 3:

**Once you have met your mentor, we ask that you share these 3 goals and that you and your mentor re-assess your success with achieving these goals after completion of each peer-reviewed manuscript.**

# Welcome Packet and Instructions

## Introduction Letter

Dear [mentor] and [mentee],

Welcome to the Mentor/Mentee Peer Review Program through the Resident & Fellow Section of *Neurology®.* This program will offer [**mentee**] the opportunity to learn the nuances of peer review through the Resident & Fellow Section (RFS) with **[mentor]**, an experienced mentor. This program will span six months and will include exactly 2 mentored reviews.

Program Format:

- A brief pre-survey was sent to mentors and mentees to determine review experience and interests
- Mentor-mentee pairs will be introduced to each other via e-mail and receive reviewing guides with helpful information
- Mentees will complete an **unassisted** baseline review of a standardized clinical manuscript. Please do your best independent work **without** help from your mentor.
- Two Mentored Manuscripts will be assigned through the *Neurology®* RFS and the request for review will include the timeline for co-reviewing which allows for additional time than a standard review. *Please add [e-mail address] to your safe list to ensure you receive these e-mails.*
- At 6 months, mentees will complete an **unassisted** review of a standardized scientific manuscript applying what they learned from their mentors
- Mentees will meet with the program coordinator at the end of the program to formally check in on the experience, discuss the unassisted review, and answer remaining questions.
- A post-program survey will be sent out at the end of the program

Reviews

- We recommend that the mentee review the manuscript and write a draft of the review and send this to your mentor prior to meeting.  The mentor should also read and review the manuscript independently prior to the meeting.
- Meet to discuss the specific manuscript and the review process.  It is helpful if the mentor can provide written feedback on the mentee’s review when appropriate.  We encourage the mentor-mentee to find a format (virtual, telephone, e-mail) that works for them.
- The mentee will submit the final review online and the mentor will respond to the review request indicating they assisted with the mentee’s review. The review should be submitted by the deadline, even if the pair are unable to meet before submitting.
- A post-review survey will be sent to the mentee following each mentored review.

If questions or concerns arise at any point during the program you should e-mail Dr. Lyons-Warren ([lyonswar@bcm.edu](mailto:lyonswar@bcm.edu)). Please find attached curated materials that you may find helpful.

Welcome to the program!

Ariel Lyons-Warren, Mentor-mentee program organizer

## Additional Resources for Peer Review

*Welcome to the mentored peer review program. We have paired you with a mentor who will co-review three manuscripts with you. We have also provided the following materials as a primer for the review process. For additional information, consider other resources such as:*

1. *Publons Reviewer Academy (*[*https://publons.com/community/academy/*](https://publons.com/community/academy/)*)*
2. *Editage Peer Reviewer Training Course (Basic- Free)* <https://wkauthorservices.editage.com/peer-reviewer-training-course/#knowmore>
3. *Blog posts such as https://www.sciencemag.org/careers/2016/09/how-review-paper or https://www.journals.elsevier.com/applied-soft-computing/news/tips-and-advice-when-you-review-a-scientific-paper*
4. *Published articles such as Rooyen et al 1999 entitled Development of the Review Quality Instrument which is a metric for reviews, see if your review scores well.*

## Structuring a Review

*Remember, you can ALWAYS reach out to fellow board members, your mentor, or myself with questions.*

**Structure of a review**

- There is no one right way to write a review.
- See appendix 1 for a sample template
- Comments to the EDITORS
  - A 1-2 sentence summary of the manuscript
  - Include strengths and weaknesses of the manuscript and a recommendation (accept, revise, reject) with support for that recommendation.
  - Don’t need to be the same as the comments to the authors
  - Comment on any ethical concerns (plagiarism, etc.)
  - Disclose any conflicts or potential biases
- Comments to the AUTHORS
  - Can be presented as major/minor concerns or by section.
  - Provide thoughtful and critical review of strengths, weaknesses, critiques and concerns
  - Provide constructive comments to help the authors and manuscript improve

## A note on tone

When writing a review, think how you would feel to read that review about your own work. The resident and fellow section is designed for trainees, and so many of our submissions come from first time authors. Therefore, comments to authors should be constructive rather than critical. It is appropriate and necessary to point out weaknesses, but always attempt to do so with a focus on teaching. Tell the authors what the problem is, why it needs to be improved, and give suggestions on how to improve it. For example, imagine a clinical reasoning case in which the question at the end of a section is “What is the diagnosis” but you think it would be better to ask “What is the differential diagnosis for altered mental status”. In this case, tell the authors that their original question is too broad. Suggest your revised question and explain that guiding the reader to think about the differential is a more effective method to teach how to approach the case.

Reviews should be well written with a focus on clarity and appropriate grammar, just as you expect from the authors.

## Factors to consider when evaluating a manuscript

- Accuracy of information: Do you agree with the final diagnosis – i.e. are the reported history, physical and lab results consistent with the diagnosis? Are the statements made in the discussion factually correct? Did you confirm that cited information can be found in the listed reference? When authors cite specific numbers from studies (i.e. 35% of patients make a good recovery) is the data from the primary paper accurately represented?
- Novelty: Has there been a prior article in the R&F section on this topic?
- Relevance: Is this a valuable topic for trainees?
- Teaching value: Does the case effectively illustrate the teaching point? Can you easily identify the take home message/key teaching point?
- Communication of ideas: Do the authors use clear, concise language that effectively communicates ideas? Are there numerous run-on sentences that obscure the message?
- Ability to address your concerns: Will it be feasible for the authors to implement changes to address your concerns, i.e. can’t go back and change what testing was done.
- Is the topic appropriate for manuscript type: If it is a clinical reasoning article, do the authors focus on the logical thought process necessary to approach that particular chief complaint? If the reasoning is not the focus, would it be more appropriate for another subsection? Did the authors follow the author guidelines for manuscript structure?
- Are all the tables and figures easy to follow, clearly labeled, and supportive of the main point the authors are trying to make?

## Appendix 1: Sample template - Comments for the editors

Thank you for the opportunity to review the manuscript: TITLE by AUTHOR et al.

Briefly, this manuscript is a [case report and discussion] submitted to the Resident and Fellow section under the category of [Clinical Reasoning]. The authors report the clinical presentation of a patient with *** and then guide the readers through ***. The manuscript ends with a ***.

The strengths of this manuscript are the [well written case presentation with a logical step-by-step assessment of how to diagnose the patient.] This type of guided evaluation is of general interest to neurology trainees. Further, *** and thus the discussion may be of interest to the full Neurology readership. *** that should be improved to provide better guided teaching as detailed in the comments for authors section. OR There are significant errors in writing and evidence of poor attention to detail resulting in lack of clarity that should be addressed prior to publication. However, overall, I think this article IS/IS NOT/COULD BE appropriate for publication in Neurology with *** revisions.

This topic is not controversial and does not need an editorial.

I do not have concerns for scientific fraud, violation of ethical treatment of human subjects, or plagiarism.

**Comments for the Authors**

The case report by AUTHOR et al, TITLE, includes *** The paper contains many excellent points, but also has many areas that could be improved to better communicate the ideas to the readers.

1. Initial patient presentation

This section provides sufficient information to guide the reader without delving into extraneous information. The authors might consider adding *** to allow the reader to consider how *** might contribute to the current presentation.

1. Section #

Comments or concerns on each section

1. General Presentation:

This paper would be improved by detailed attention to grammar. Streamlining of sentence structure could shorten the article and allow room for addressing some of the points of interest mentioned above.

Limit your comments to 300-600 words. Do not edit the paper for grammar, spelling, etc., although you may comment that these need improvement. All your comments should be objective and correct, intended to help the author improve the paper. Comments should be courteous, constructive, never personal or offensive.

## Appendix 2: Evaluating your review

From Susan van Rooyen, Nick Black, and Fiona Godlee (1999) Development of the Review Quality Instrument (RQI) for Assessing Peer Reviews of Manuscripts. J Clin Epidemiol Vol. 52, No. 7, pp. 625–629.

1. Did the reviewer discuss the importance of the research question?

1 2 3 4 5

Not at all Discussed extensively

2. Did the reviewer discuss the originality of the paper?

1 2 3 4 5
Not at all Discussed extensively with references

3. Did the reviewer clearly identify the strengths and weaknesses of the method (study design, data colletion and data analysis)?

1 2 3 4 5
Not at all Comprehensive

4. Did the reviewer make specific useful comments on the writing, organization, tables and figures of the manuscript?

1 2 3 4 5
Not at all Extensive

5. Were the reviewer’s comments constructive?

1 2 3 4 5
Not at all Very constructive

6. Did the reviewer supply appropriate evidence using examples from the paper to substantiate their comments?

1 2 3 4 5
No comments substantiated All comments substantiated

7. Did the reviewer comment on the author’s interpretation of the results?

1 2 3 4 5
Not at all Discussed extensively

## Mentor Evaluation of Reviews

*Thank you again for agreeing to* ***mentor*** *a resident on the peer review process. These materials are designed to make mentoring easier for you and more standardized for all mentees. Please feel free to e-mail me with any questions or suggestions.*

**2 co-reviewed manuscripts**

For each manuscript you co-review with your mentee, please evaluate for clarity, helpfulness, thoroughness, and accuracy.

- 1. Clarity
     1. Did the trainee clearly communicate to the authors the areas that need to be improved?
     2. Did the trainee’s comments have an appropriate, professional tone?
     3. Was the review organized logically?
  2. Helpfulness
     1. Were the suggestions implementable?
     2. Were the comments constructive rather than just critical?
     3. Did the comments to the editor include strengths, weaknesses and a recommendation to allow the editor to make an informed decision? Did the comments to the author clearly identify those same strengths and weaknesses?
  3. Thoroughness
     1. In the comments for editors did the trainee mention/consider originality, relevance or importance, teaching value, communication of ideas, ability to address concerns, appropriateness for manuscript type, and accuracy of information?
     2. In the comments for authors, did the trainee evaluate each section, table and figure? Was there anything you were concerned about that the trainee did not mention?
     3. Did the trainee check references to ensure that the cited information is contained in the referenced publication?
     4. Did the trainee substantiate their comments with examples.
  4. Accuracy
     1. Do you agree with the trainee’s recommendation regarding publication?
     2. Do you agree with the trainee’s assessment of the author’s conclusion(s)?

You can also consider using the Review quality Instrument which is an 8 question assessment of the quality of a review^[[1]](#footnote-1)^.

The amount of feedback will depend on the mentee’s prior reviewing experience. For the first co-review, we recommend that you read the manuscript and prepare your own review. When reading the mentee’s review, provide written or verbal feedback using the questions above as a guide. It is okay to share your review with the mentee so that they can learn by example. Read through the mentee’s review one last time prior to submission to see how they incorporated your feedback and provide 2-point feedback on the final version. In 2-point feedback, highlight one area the mentee improved on by following prior suggestions well and one area the mentee can focus on for the next review.

On the second and third co-review the mentee maybe able to function more independently. Consider using each of these reviews as a discussion point for common mistakes such as:

- - - Inconsistent recommendation: final recommendation does not fit with degree of comments
    - Vague comments: Comments to authors are not specific enough such as “unclear” or “needs work” without explaining what the underlying problem is
    - Too nice: Due to inexperience, new reviewers may be reluctant to point out problems

Or have a helpful discussion on a topic such as review structure (major/minor concerns vs by section), what makes a good teaching case, or how to evaluate an opinion article.

# Mentee First Review Follow-up Survey

**Great work completing your FIRST mentored review in the Resident & Fellow Section of Neurology®. The following questions will help us to develop and optimize this program for our mentors and your fellow mentees. We ask that you please complete the following short questions which we will use as we continue to develop this program. Thank you for your participation.**

Name: _______________ Date: _________

Please answer the following questions:

1. Were you able to meet with you mentor to complete your review (select one)?

No

Yes, one time

Yes, multiple times

2. Did you meet with your mentor before and/or after writing your review (select one)?

Before

After

Both

3. What was one thing you learned from your mentor while completing this review?

4. What is one way in which you will change your approach to future reviews based on this experience?

5. Do you have any questions or concerns about the program you would like us to follow up with you about?

# Mentee Second Review Follow-up Survey

**Thank you for completing your SECOND mentored review in the Resident & Fellow Section of Neurology®. The following questions will help us to develop and optimize this program for our mentors and your fellow mentees. We ask that you please complete the following short questions which we will use as we continue to develop this program. Thank you for your participation.**

Name: _____**_______ ___**____________ Date: **______**

Please answer the following questions:

1. Were you able to meet with you mentor to complete your review (select one)?

No

Yes, one time

Yes, multiple times

2. Did you meet with your mentor before and/or after writing your review (select one)?

Before

After

Both

3. What was one thing you learned from your mentor while completing this review?

4. What is one way in which you will change your approach to future reviews based on this experience?

5. Do you have any questions or concerns about the program you would like us to follow up with you about?

6. Overall, do you feel that the mentored-review program improved your ability to produce a clearly written review that provides useful guidance to editors and authors?

Please answer the following questions by selecting a rating:

7. I understand the review process involved in peer review of scientific manuscripts:

1  2  3  4  5

Strongly disagree Disagree Neutral Agree Strongly agree

8. I understand the editorial process involved in processing a manuscript for publication:

1  2  3  4  5

Strongly disagree Disagree Neutral Agree Strongly agree

9. I am confident that I can independently review a SCIENTIFIC manuscript at a high level:

1  2  3  4  5

Strongly disagree Disagree Neutral Agree Strongly agree

10. I am confident that I can independently review a NON-SCIENTIFIC (i.e. clinical) manuscript at a high level:

1  2  3  4  5

Strongly disagree Disagree Neutral Agree Strongly agree

## Mentor Survey regarding Mentor/ Mentee Program

1. I enjoyed the opportunity to review Resident & Fellow Section manuscripts with my mentee?

☐​ 1 ​☐​ 2 ​☐​ 3 ​☐​ 4 ☐​ 5

Strongly disagree Disagree Neutral Agree Strongly agree

1. I would consider participating in this program again in the future? 
   ☐​ 1 ​☐​ 2 ​☐​ 3 ​☐​ 4 ​☐​ 5

Strongly disagree Disagree Neutral Agree Strongly agree

1. What barriers, if any, did you have when trying to meet with your mentee?
2. What was the biggest impact to *you* of participating in this program?
3. Do you have any suggestions for how to improve the program?

# Modified Review Quality Index

**MS ID: Referee ID: Editor ID:**

This review contains:

1. A summary of the study at the beginning of the review. YES / NO

2. Separate comments for editors and for authors. YES / NO

3. Comments divided as major/minor or section-wise. YES / NO

4. A formal recommendation as to acceptance/rejection. YES / NO

1. Susan van Rooyen, Nick Black, and Fiona Godlee (1999) Development of the Review Quality Instrument (RQI) for Assessing Peer Reviews of Manuscripts. J Clin Epidemiol Vol. 52, No. 7, pp. 625–629. [↑](#footnote-ref-1)
